# Supplementary material for: The Phylogeny, Metabolic Potentials, and Environmental Adaptation of an Anaerobe, Abyssisolibacter sp. M8S5, Isolated from Cold Seep Sediments of the South China Sea
Source: Microorganisms. 2023 Aug 25;11(9):2156. doi: 10.3390/microorganisms11092156 (PMC10536192; doi:10.3390/microorganisms11092156)
Supplement: Supplementary file 1 [file microorganisms-11-02156-s001.zip › Supplementary Figure S1.pdf]

Legends of supplementary figures

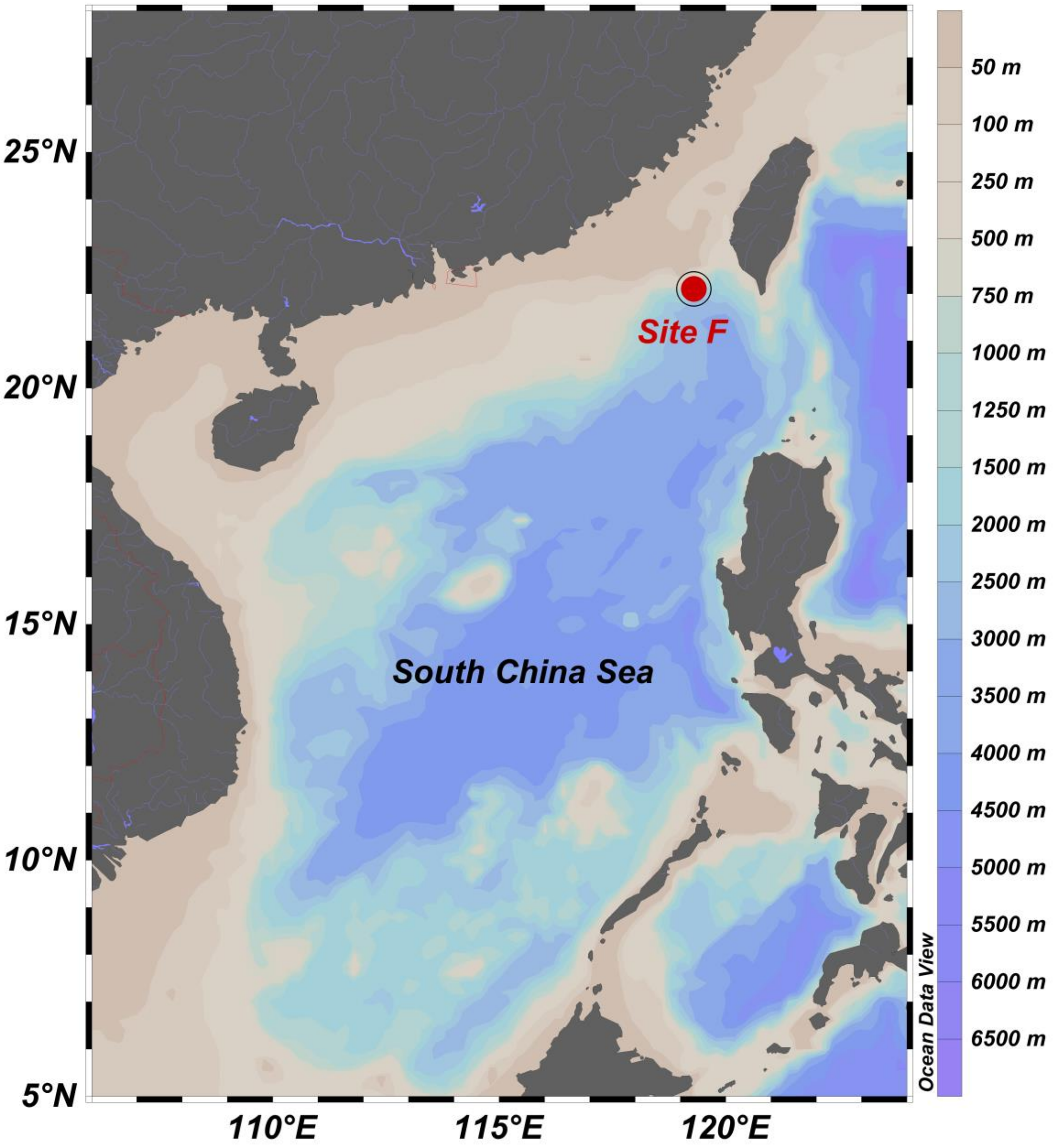

Supplementary Figure S1. Sampling locations of the active cold-seep (Site F) in the South China Sea. Site F is highlighted by red circles.
